# Case Report: Cronkhite-Canada syndrome: presentation of a pediatric case and review of the literature

**DOI:** 10.3389/fped.2024.1451472

**Published:** 2024-09-24

**Authors:** Weina Shi, Haiyan Fu, Shiguang Zhao, Shuhuan Cheng, Shaogang Hou, Ruiqin Zhao

**Affiliations:** Department of Gastroenterology, Children’s Hospital of Hebei Province, Shijiazhuang, Hebei, China

**Keywords:** Cronkhite-Canada syndrome, diarrhea, gastrointestinal polyposis, alopecia, skin hyperpigmentation, children

## Abstract

**Background:**

Cronkhite-Canada syndrome (CCS) is extremely rare in children, presenting with complex clinical manifestations often leading to misdiagnosis.

**Case presentation:**

We reported a description of a 13-year-old boy with CSS presenting with persistent diarrhea, vomiting, abdominal pain, along with symptoms of weight loss, alopecia, and skin hyperpigmentation. The patient had ectodermal manifestations such as alopecia and skin hyperpigmentation. Laboratory tests revealed hypoalbuminemia, normal inflammatory indicators, positive anti-dsDNA antibodies, anti-centromere antibodies, and anti-nuclear antibodies. Gastrointestinal endoscopy identified polypoid changes in the stomach, duodenum, and colon, with pathology indicating glandular dilation, cryptitis, and crypt abscesses. Treatment with prednisone led to significant improvement in symptoms, including normalization of stool consistency, hair regrowth, and disappearance of skin hyperpigmentation.

**Conclusion:**

This study emphasizes the importance of comprehensive assessment, endoscopic examination, histological biopsy, and the effectiveness of steroid therapy in the diagnosis and management of CCS in children. In children presenting with diarrhea, abdominal pain, weight loss, polyposis, and ectodermal manifestations, CCS should be considered.

## Background

Cronkhite-Canada Syndrome (CCS), also known as polyposis-pigmentation-alopecia-onychodystrophy syndrome, is a rare non-genetic disease characterized by abdominal pain, diarrhea, weight loss, protein-losing enteropathy, extensive gastrointestinal polyps, and ectodermal lesions (including alopecia, skin pigmentation, and onychodystrophy) (1). CCS typically occurs in middle-aged or elderly individuals. A retrospective study in Japan found that the average age at diagnosis was 63.5 years (2). A Chinese report summarized over 100 patients, with onset ages ranging from 9 to 82 years old, and 62.62% of them were between 50 and 70 years old (3). Reports of pediatric cases are extremely rare, making it particularly important to explore the clinical features, pathophysiological mechanisms, and optimal treatment strategies for patients in this age group. This study aimed to delve into the clinical characteristics of a pediatric CCS case presenting primarily with diarrhea. By reviewing existing literature, we aimed to broaden the understanding of the clinical spectrum of pediatric CCS and guide clinical practice. The diagnostic journey of this patient not only highlighted the complexity of CCS diagnosis but also emphasized the importance of early identification and therapeutic intervention, especially when facing diarrhea, ectodermal manifestations, and ineffectiveness of conventional treatments. Through comprehensive analysis, we hoped to provide valuable insights to clinicians, reducing missed and misdiagnoses while promoting deeper research into the mechanisms of this unique syndrome.

## Case report

### Chief complaint and present illness

The patient was a 13-year-old male who presented with diarrhea 2 weeks ago with no apparent cause, accompanied by vomiting and abdominal pain. Diarrhea occurred 6–7 times a day. Abdominal pain mainly worsened after eating. In addition, the patient had joint discomfort in both knees and left big toe. Within two weeks, the child lost 2.5 kg. No fever, rash, or mouth sores. The patient was first seen at a local hospital. The results of routine stool examination in the community hospital showed increased white and red blood cell counts in the stool [16–25 /High power field (HPF) and 55–72 /HPF, respectively]. The patient was treated with cefoperazone and Saccharomyces boulardii sachet in local hospital. However, the symptoms of diarrhea continued to worsen, accompanied by dark red bloody stool, and gradually developed symptoms of hair loss. Subsequently, the patient came to our hospital for treatment, and was admitted with acute bacterial gastroenteritis. The child has no history of special diseases, no family history of heredity, and no history of exposure to infectious diseases.

### Physical examination

**Figure 1:**
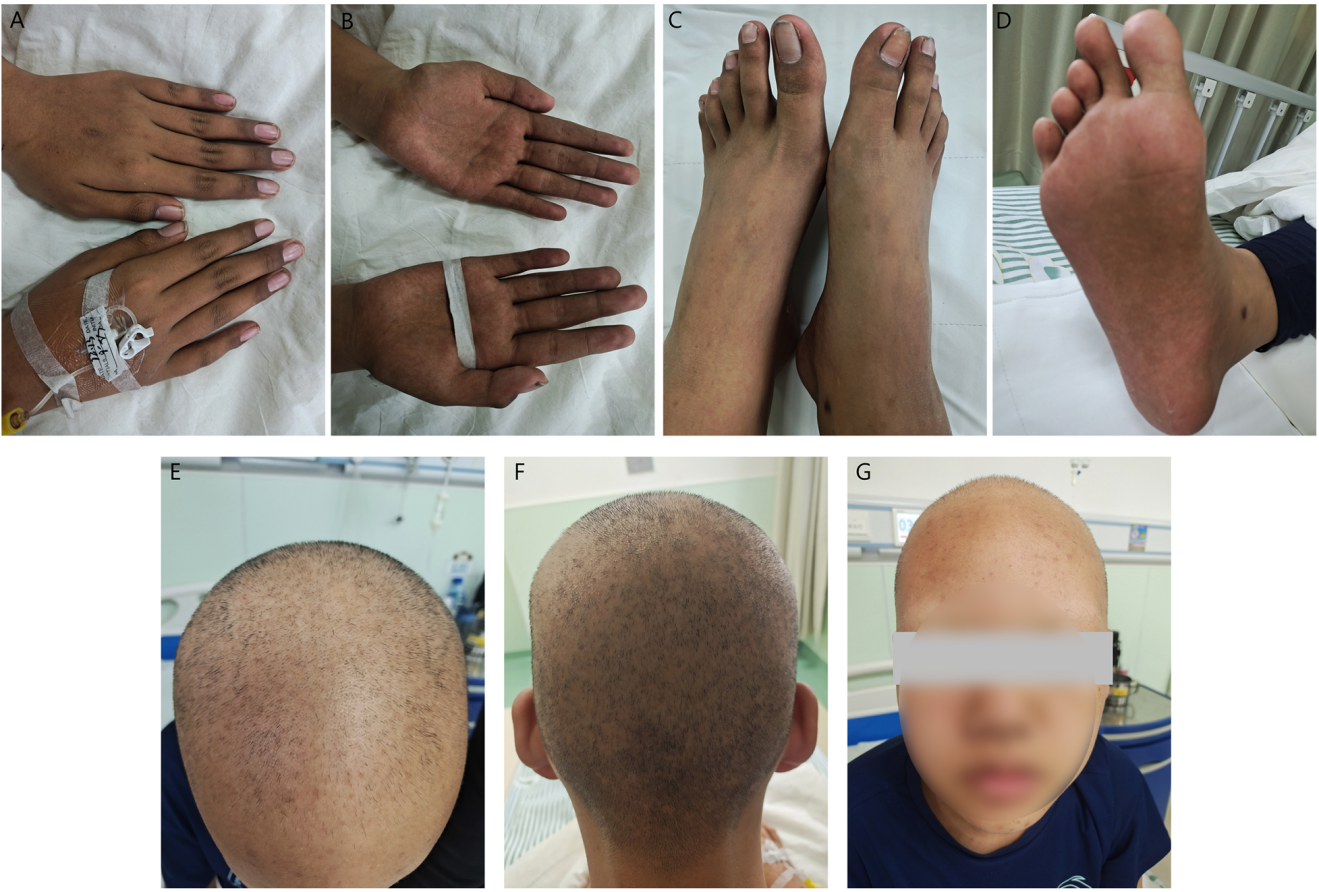
Clinical presentation before treatment **(A)** pigmentation on the dorsum of both hands; **(B)** pigmentation on the palms of both hands; **(C,D)** pigmentation on both feet; **(E,F)** hair loss; **(G)** loss of eyebrows.

Physical examination on admission: the patient was conscious. There was extensive scattered acne on the face, and hair and eyebrows fell out. Skin pigmentation on both hands and feet. (Figure 1). Abdominal examination did not reveal liver or spleen.

### Laboratory, imaging and endoscopy results

Blood routine examination: eosinophils decreased by 0.04% (0.5%–5.0%). Liver function test: Total protein decreased by 57.5 g/L (68–88 g/L), albumin decreased by 40.3 g/L (42–56 g/L), globulin decreased by 17.2 g/L (19–40 g/L). Fecal calcium protective protein was increased by 975 ug/g (0–200 ug/g). Indicators of infection: normal C-reactive protein (CRP), normal erythrocyte sedimentation rate (ESR). Procalcitonin (PCT) was increased by 0.09 ug/L (0–0.06 ug/L). Other blood tests showed no significant abnormalities, including electrolytes, liver function, renal function, myocardial enzymology, serum amylase, thyroid function, trace elements, adrenocorticotropin (ACTH), Helicobacter pylori antibody, and autoantibody tests. No bacterial and fungal growth and no clostridium difficile toxins were observed in fecal culture.

**Figure 2:**
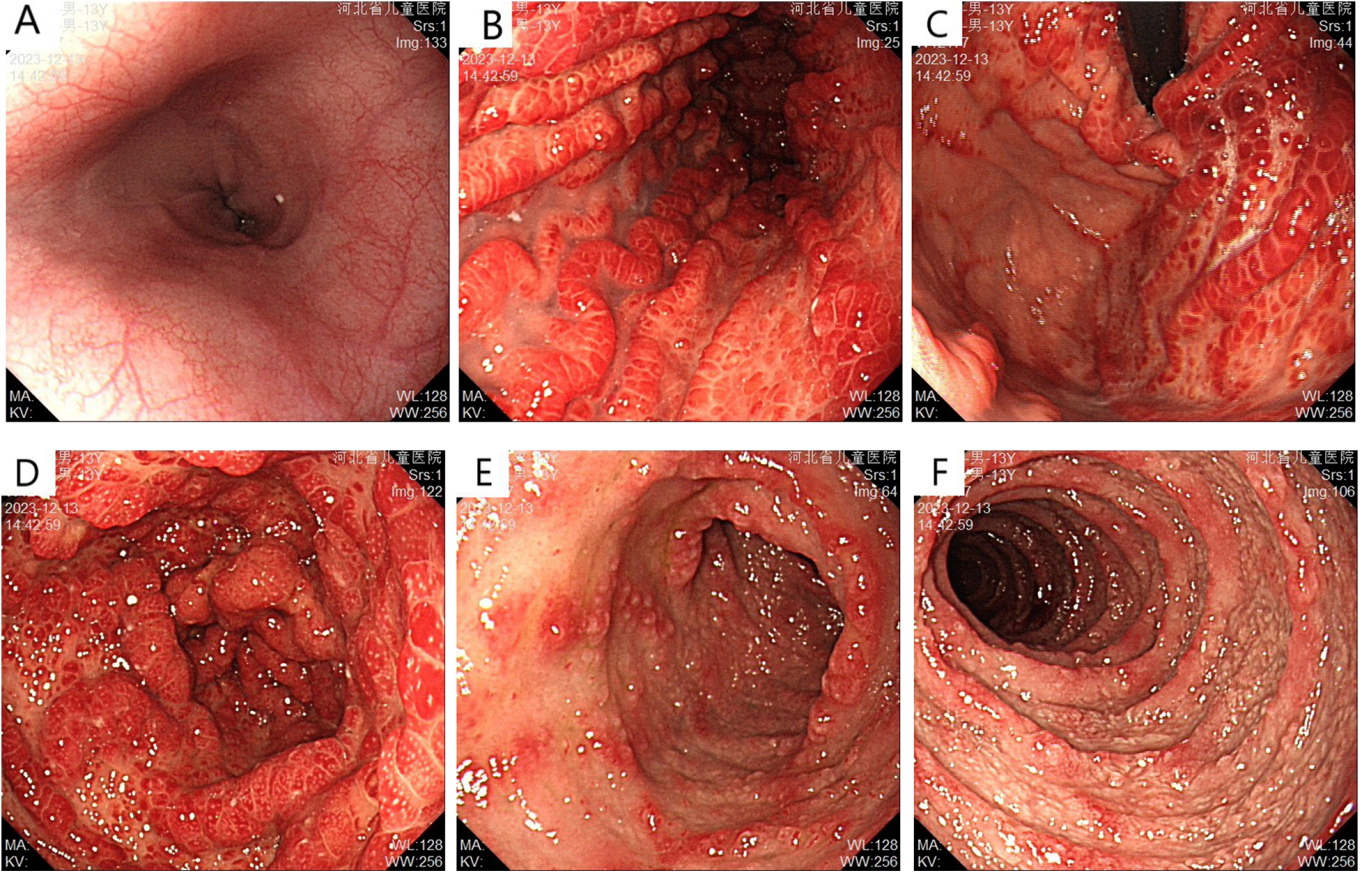
Upper gastrointestinal endoscopy before treatment **(A)** normal esophageal mucosa; **(B)** gastric folds are thickened with diffuse congestion and edema in the body; **(C)** diffuse congestion and edema with nodular changes in the fundus mucosa; **(D)** diffuse congestion and edema with nodular changes, some presenting as “mulberry-like” appearance; **(E,F)** multiple nodular changes with erosion observed in the duodenal bulb and descending part.

**Figure 3:**
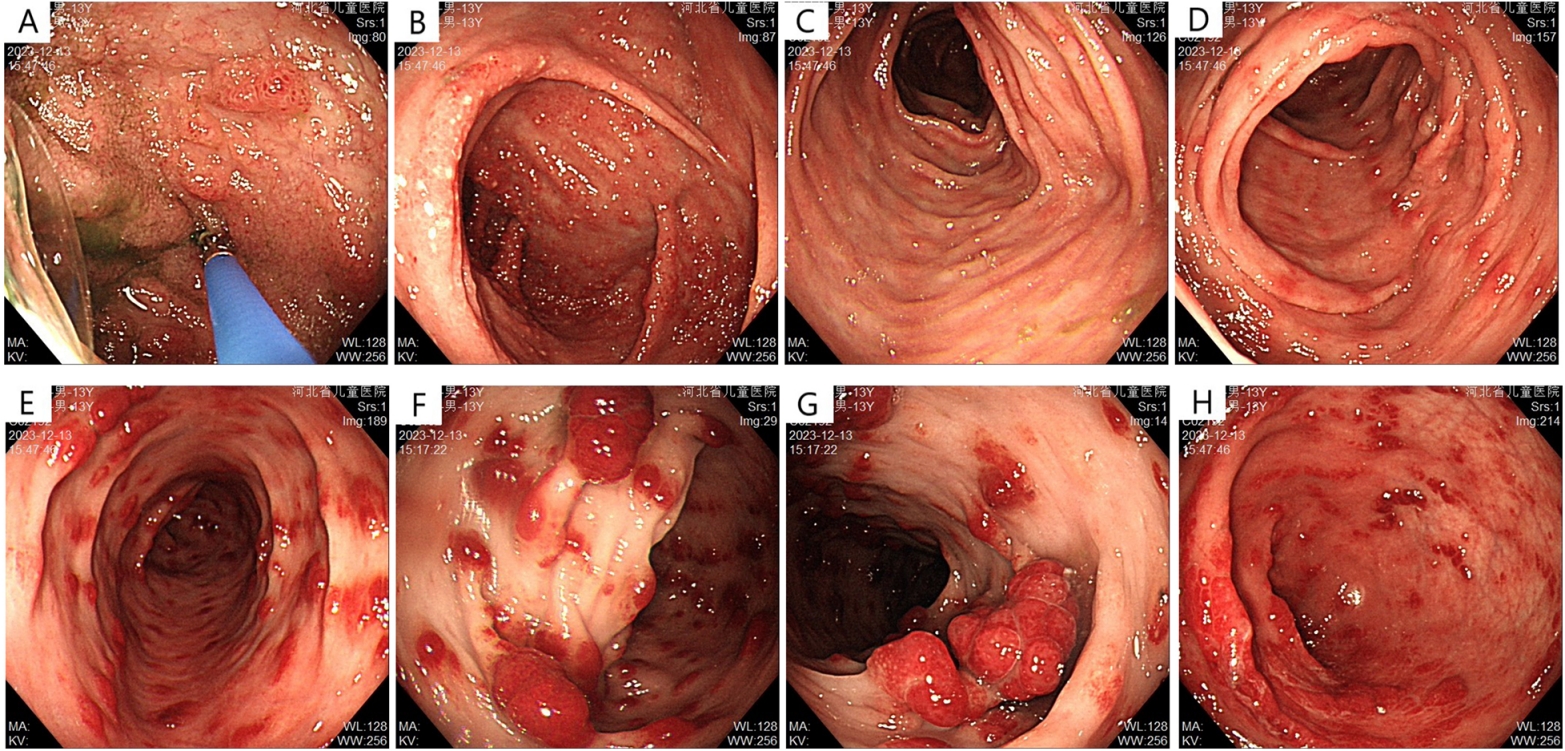
Lower gastrointestinal endoscopy before treatment **(A)** terminal ileum: scattered nodular changes; **(B)** cecum: dense nodular elevations with apical erosion; **(C,D)** patchy erosions visible in the ascending and transverse colon; **(E)** descending colon: polypoid elevations, “strawberry-like” change, sessile; **(F,G)** multiple polypoid elevations in the sigmoid colon, “strawberry-like” change, mostly sessile; **(H)** multiple polypoid elevations in the rectum, sessile.

**Figure 4:**
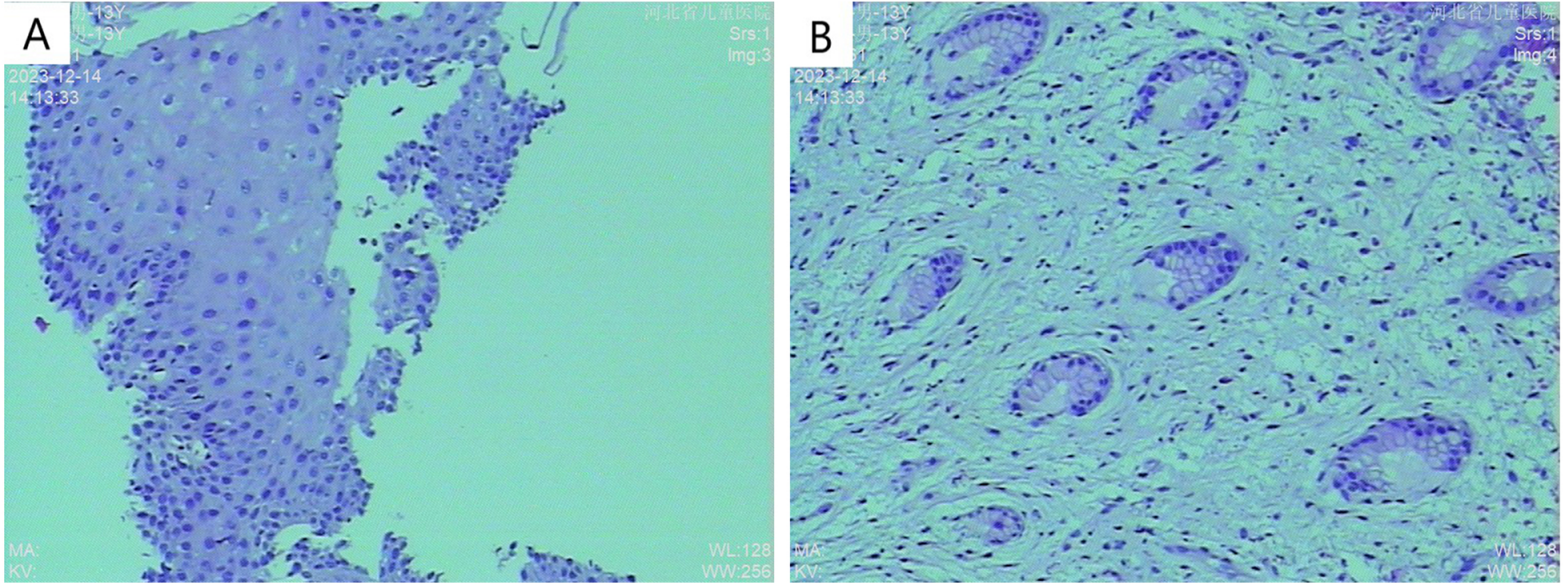
Histopathological examination **(A)** H&E stain, 10× magnification showing partial glandular dilation; **(B)** H&E stain, 10× magnification revealing glandular dilation, cryptitis, and crypt abscesses.

Abdominal ultrasonography showed no significant abnormalities. Gastrointestinal endoscopy showed that the esophageal mucosa was normal, with multiple nodules or polypoid changes in the stomach, duodenum, and colon. Pathological changes of some intestinal segments suggested inflammatory changes, glandular dilatation, and eosinophilia (such as ileocecal valve, ascending colon, and ileum) (Figures 2–4). The skin microscope showed abnormal hair.

### Final diagnosis and treatment

Based on clinical findings, laboratory tests, imaging, endoscopy, and pathological findings, the final diagnosis was Cronkhite-Canada syndrome. The patient was given prednisone intravenously at 50 mg/day for 8 weeks. At the same time, montmorillonite powder was given to protect the gastrointestinal tract, probiotics to regulate the flora, and rehydration therapy. Subsequently, the patient's symptoms gradually improved, including diarrhea ceased, weight gradually gained, hair regrowth, and pigmentation disappeared (Supplementary Figure S1). Subsequently, the dosage of prednisone was gradually reduced by 5 mg/day every 2 weeks. When the duration of prednisone treatment reached 20 weeks, the patient's symptoms were completely resolved.

## Literature review

A literature search was conducted using the keyword “Cronkhite-Canada Syndrome” in PubMed database from establishment to May 2024. Additionally, using the keywords “Cronkhite-Canada Syndrome” and “Polyp-Pigmentation-Alopecia-Nail dystrophy syndrome” in the Chinese Journal Full-text Database and CNKI database from establishment to May 2024, domestic literature was retrieved.

8 pediatric cases were identified in the literature (4–11), involving a total of 8 patients (Table 1). 50% of the patients were infants, the ratio of males to females was 3.5:1, and the age of onset was from 4 months to 17 years. All patients had diarrhea, extensive polyps in the gastrointestinal tract, and at least one ectodermal lesion (alopecia, nail dystrophy, or pigmentation). All the infant CCS patients had giant malformation and clubbing fingers, and 3 of them died after surgical treatment. The causes of death were lung infection, heart failure and cardiac arrest.

**Table 1:** Case reports summary of 8 CCS patients.

Case no.	Patient age	Gender	Origin	Clinical manifestations	Polyp pathology	Year of publication	Family history	Prognosis
1	5 months	Female	Sri Lanka	Diarrhea with mucus and blood, anemia, hypoproteinemia, macrocephaly, multiple intestinal and colonic polyps, clubbing of fingers, alopecia	Juvenile polyps	1997	Negative	Surgical intervention, deceased several months post-treatment
2	6 months	Male	South Africa	Diarrhea with mucus and blood, anemia, hypoproteinemia, hepatosplenomegaly, macrocephaly, hypotonia, gastric, duodenal, intestinal, and colonic polyps, alopecia, clubbing of fingers, nail dystrophy, biventricular heart failure	Juvenile polyp hamartoma	1986	Unknown	Surgical intervention, deceased at 14 months
3	2 years old	Female	Turkey	Bloody stool, hepatosplenomegaly, clubbing of fingers, anemia, macrocephaly, abdominal distension, diffuse gastrointestinal polyps	Juvenile polyps	1992	Negative	Nutritional management, condition improved after 13 months of follow-up
4	15 years old	Male	Brazil	Watery diarrhea, vomiting, abdominal pain, weight loss, skin pigmentation, nail dystrophy, hypoproteinemia, extensive gastric, duodenal, and colonic polyps	Not specified	2018	Unknown	Symptomatic improvement after 9 months of hormone therapy and follow-up
5	17 years old	Male	Italy	Watery and bloody stool, weight loss, abdominal pain, anorexia, alopecia, skin pigmentation, nail dystrophy, hypoproteinemia, extensive gastric, duodenal and colonic polyps, Type I diabetes, thalassemia, concomitant diffuse membranous nephropathy	Juvenile hyperplastic polyps	2005	Negative	Improved with nutritional management, asymptomatic after 7 years of cyclosporine follow-up
6	9 months	Male	Polynesia	bloody diarrheal stools, hepatosplenomegaly, clubbing of fingers, anemia, macrocephaly, abdominal distension, alopecia, hypotonia, ascites, diffuse gastrointestinal polyps	Juvenile polyps	1969	Negative	Surgical intervention, deceased at 16 months
7	16 years old	Male	Korea	Hematochezia, diarrheal onychodystrophy edema of face and lower extremities duodenal and colonic polyps anemia, hypoproteinemia, hypokalemia	Hamartomatous polyps with cystic dilatation of glands	1988	Negative	Blood transfusion, albumin infusion, iron tablet medication follow-up
8	9 years old	Male	China	Abdominal pain, diarrhea and bloody stools, multiple polyps in stomach, duodenum, cecum and colon	Unkown	2020	Positive his mather mutation of APC gene	Mesalazine (0.25 g orally t.i.d) The symptoms released, but polyps burden did not reduce significantly

CCS, Cronkhite-Canada syndrome.

Kucukaydin reported a case of a 2-year-old child with CCS, which presented as bloody stool, hepatosplenomegaly, clubbing of fingers, anemia, macrocephaly, abdominal distension, diffuse gastrointestinal polyps (8). Gastrointestinal polypectomy, total colectomy, ileostomy, postoperative anti-infection and total parenteral nutrition were followed up for 13 months. Hematochezia stopped and anemia was gradually corrected. A 17-year-old CCS patient reported by Vernia P et al. (5) manifested as watery and bloody stool, weight loss, abdominal pain, anorexia, alopecia, skin pigmentation, nail dystrophy, hypoproteinemia, extensive gastric, duodenal and colonic polyps. When the high-calorie, high-protein diet with vitamin and micronutrient oral supplementation was given, the hemoglobin of the patient returned to normal and the ectodermal symptoms gradually disappeared. Subsequently, it was found that the patient had membranous nephropathy, and the serum protein was still lower than normal. The patient was given cyclosporine and other immunosuppressants, and the serum protein of the child returned to normal. After 7 years of follow-up, there were no gastrointestinal symptoms such as abdominal pain and hematochezia.

Another study (4) reported a 13-year-old male child, watery diarrhea, vomiting, abdominal pain, weight loss, skin pigmentation, nail dystrophy, hypoproteinemia, extensive gastric, duodenal, and colonic polyps were the main symptoms. After treatment with hormones and metronidazole, the child gained weight. After 160 days of hormone therapy, the child's symptoms were completely resolved, but after 5 days of discontinuation, diarrhea and skin symptoms reappeared. Prednisone 20 mg/day was given again, and the symptoms continued to improve. Kang, YW et al. (10) reported a 16-year-old child with Peutz-Jeghers syndrome with CCS-related symptoms such as onychodystrophy, edema of face and lower extremities, but his father and sister had no relevant symptoms. After treatment with blood transfusion, albumin infusion and iron tablet medication, the symptoms of skin pigmentation and edema disappeared. In summary, all adolescent patients had watery diarrhea, weight loss and skin pigmentation. Hormone, immunosuppressive, nutritional therapy and other measures can improve the symptoms, and the prognosis was better for infants. Most cases had no familial genetic tendency, but Ning, H et al. (11) reported a case of cronite-Canada Syndrome, which suggested a familial tendency. The mother was 31 years old and the son was 9 years old. Genetic test found that both of them had the same APC gene mutation. After the treatment of mesalazine, diarrhea, abdominal pain, hematochezia and other symptoms improved.

## Discussion

The CCS is a clinically rare disease characterized by the dual features of widespread gastrointestinal polyps and ectodermal abnormalities, posing challenges in the diagnosis and treatment strategies. In light of this complexity, it is crucial to deepen the understanding of CCS and expand discussions based on the latest academic advancements.

The pathogenesis of CCS remains unclear, but its close association with the immune system is becoming increasingly evident. Studies have indicated a potential role of autoimmunity in the pathophysiology of CCS, as evidenced by the co-occurrence of various autoimmune diseases such as primary hypothyroidism, membranous nephropathy, and the presence of autoantibodies like antinuclear antibodies and anti-centromere antibodies (12). Furthermore, the discovery of IgG4 plasma cells, variations in specific genes such as FDFT1, LOC400863, and the impact of serotonin-mediated intestinal epithelial proliferation (13, 14) collectively paint a complex picture of multifactorial pathogenesis in CCS. While these genetic and immunological findings vary, they undoubtedly pave the way for precision medicine in the future.

Diagnosis of CCS lacks a gold standard and relies on a comprehensive analysis of detailed medical history, physical examination, endoscopy, and pathology (15). In the case of this pediatric patient, the diagnostic process involved ruling out conditions such as Peutz-Jeghers syndrome and hereditary polyposis, ultimately confirming the diagnosis based on characteristic gastrointestinal symptoms, ectodermal changes, and the endoscopic finding of “currant jelly-like” polyps in the gastrointestinal tract, consistent with literature descriptions (16). Notably, the presence of cryptitis and abscesses in this case highlights the need for differentiation from inflammatory bowel disease (IBD). While the elevated fecal calprotectin aligns with the inflammatory nature of CCS, the distinction from IBD is crucial. In this case, the elevated fecal calprotectin suggests an inflammatory gastrointestinal disease related to the immune system, consistent with the findings of Justin Wenhao Leong et al. (17). Additionally, the significant infiltration of eosinophils in the gastrointestinal mucosa, as indicated by pathology, aligns with literature reports (18), although differentiation from eosinophilic gastrointestinal diseases is warranted. The diagnosis of eosinophilic gastrointestinal diseases is based on clinical symptoms and histological findings of eosinophilic inflammation following the exclusion of secondary causes or systemic diseases. Evaluation of other clinically relevant conditions associated with increased mucosal eosinophils is necessary before a diagnosis can be made solely based on histology (19). In this case, the presence of gastrointestinal symptoms and ectodermal changes, combined with endoscopic and pathological features, leans towards a diagnosis of CCS.

Treatment strategies for CCS are diverse and lack consensus. Corticosteroids are commonly used as the first-line treatment, leading to significant symptom improvement in this case, akin to cases of long-term remission reported in the literature (20, 21). However, the issue of relapse upon steroid tapering or cessation underscores the importance of treatment continuity and monitoring. For refractory cases, attempts with immunosuppressants or anti-TNF-α agents have been reported (22). Traditionally, the prognosis of CCS has been poor, but with advances in treatment, reports such as the long-term survival rates from the Mayo Clinic showing a 5-year overall survival rate of 93.3% and a three-year recurrence-free survival rate of 82.4% demonstrate improved outcomes (23). Long-term follow-up of this pediatric CCS case will help fill gaps in understanding the prognosis for children.

In conclusion, the case report and literature review reminded CCS of its existence as a rare disease and raised awareness about the diagnosis and treatment of the disease.

## Data Availability

The original contributions presented in the study are included in the article/Supplementary Material, further inquiries can be directed to the corresponding author.

## Supplementary material

Supplementary Figure 1 Clinical presentation after treatment **(A)** regrowth of eyebrows; **(B)** regrowth of scalp hair; **(C)** disappearance of pigmentation on the dorsum of hands; **(D)** disappearance of pigmentation on the palms.
