# Supplementary figures and images for: Case Report: Cronkhite-Canada syndrome: presentation of a pediatric case and review of the literature
Source: Front Pediatr. 2024 Sep 24;12:1451472. doi: 10.3389/fped.2024.1451472 (PMC11458448; doi:10.3389/fped.2024.1451472)

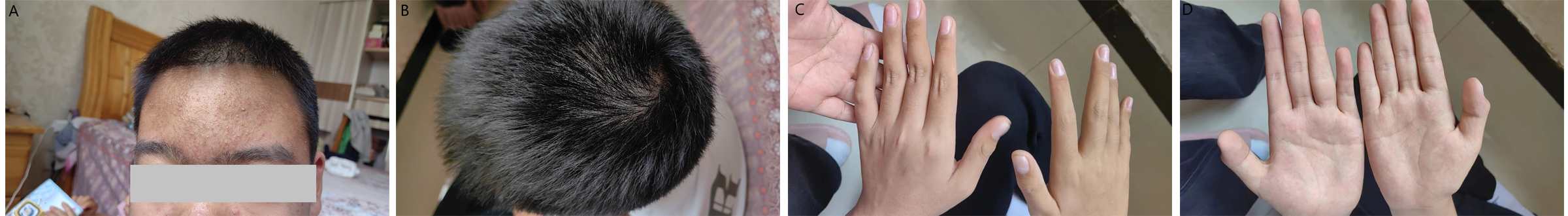

Supplement: Supplementary Figure 1 — Clinical presentation after treatment (A) regrowth of eyebrows; (B) regrowth of scalp hair; (C) disappearance of pigmentation on the dorsum of hands; (D) disappearance of pigmentation on the palms. [file Image1.jpeg]
